# Associations of Education Level and Bone Density Tests among Cognitively Intact Elderly White Women in Managed Medicare

**DOI:** 10.1155/2012/179150

**Published:** 2012-09-26

**Authors:** Di Shi, Michael T. Yin, Qiuhu Shi, Donald R. Hoover

**Affiliations:** ^1^School of Medicine and Biomedical Sciences, University at Buffalo, 131 Biomedical Education Building, Buffalo, NY 14214, USA; ^2^Division of Infectious Diseases, Columbia University Medical Center, PH8-876, 630 w168th Street, New York, NY 10032, USA; ^3^Department of Epidemiology and Community Health, School of Health Sciences and Practice, New York Medical College, Valhalla, New York, NY 10595, USA; ^4^Department of Statistics and Institute for Health, Health Care Policy and Aging Research, Rutgers University, 110 Frelinghuysen Road, Room 473, Piscataway, NJ 08854, USA

## Abstract

*Objectives*. To examine associations between having bone density tests and level of education among white elderly women in managed Medicare. *Method*. Data from the ninth through twelfth cohort (2006–2009) of the Medicare Health Outcome Survey (HOS) of managed Medicare plans were analyzed; 239331 white elderly women were included. Respondents were grouped by education level and the percentages of respondents who had lifetime bone density testing done among each group were analyzed. *Results*. 62.7% of respondents with less than a high school education reported previously taking a bone density test. This was lower than the 73.8% for respondents who completed high school and the 81.0% for respondents with more than a high school education. When potential confounding factors such as age, body mass index, marital status, smoking history, year of HOS survey, and region were factored in, the odds ratios of having a bone density test when compared to respondents with less than a high school education were 1.61 and 2.39, respectively, for those with just a high school education and more than a high school education (*P* < 0.001). *Conclusion*. Higher education was independently associated with greater use of bone density test in these elderly white women.

## 1. Introduction

In recent years, many studies have found a link between education level and the overall health of a person. Lower education was found worldwide to be correlated with increases in many areas of health risk including cardiac dysfunction [1], preterm birth [2], mortality [3], and others diseases [4, 5]. Furthermore, these effects of education disparity on greater disease prevalence [4] and lower life expectancy may be growing [6]. When treating patients with lower education levels, physicians spend less time discussing health related issues [7] which may lead to gaps in health awareness including the use of preventive services. For example, lower levels of education have been linked to lower use of public health services in Brazil [8] and lower use of HIV testing in Portugal's immigrant population [9]. The goal of this paper is to see if lower education also effects use of bone mineral density test among a group that would benefit most from testing, elderly white women in the United States.

Bone fractures can have devastating consequences including pain, loss of mobility function, and death. Among elderly, particularly postmenopausal women, deterioration in bone mass progressively increases the risk of fragility fractures compared to people with healthy bone [10]. While all elderly women in America are at higher risk for fractures, Caucasians are at higher risk than others in particular African Americans. Among 50-year-old white women, the risk for suffering a hip fracture at some future point in their life is 16% while the risks for suffering a forearm or thoracic/lumbar spine fracture are 15% and 32%, respectively, [11]. Hip fractures are particularly devastating; an estimated 24% of hip fracture patients die within one year of their admission to a hospital [12].

The risk for fractures dramatically rises with osteoporosis. Osteoporosis is defined as having bone mineral density (BMD) of more than 2.5 standard deviations below the mean peak bone mass average of young, healthy adults as measured by the dual-energy X-ray absorptiometry (DXA or DEXA) scan [13, 14]. Effective treatments to prevent fractures include calcium/vitamin D supplementation, increased exercise, smoking cessation, and a variety of medical agents including bisphosphonates, estrogen analogs, and bone anabolic agents [15–19].

Despite the risks for and consequences of osteoporosis particularly in women, many elderly women do not get a bone density test. The aim of this paper is to explore how education level of cognitively intact elderly women contributes to nonuse of this preventive service. We focus on elderly white women because this group is at a particularly high risk for osteoporosis and fracture and also to eliminate the effects of any health disparities that might arise due to race and gender. The data used are from the ninth through twelfth cohorts of the Medicare Health Outcomes Survey (HOS) which collects information from elderly people receiving managed Medicare. These four cohorts were chosen because the bone density test question only existed in these cohorts.

## 2. Methods

### 2.1. Data Source and Study Design

This study used data from the Medicare Health Outcomes Survey (HOS). The HOS is a publically available longitudinal cohort survey conducted by the Centers for Medicare & Medicaid Services (CMS) to evaluate the quality of managed Medicare Advantage Organizations (MAO). Every year, the HOS conducts a baseline survey on 1200 randomly chosen members of each organization (i.e., managed plan). More details on the HOS are available on the internet at http://www.hosonline.org/Content/Default.aspx. This paper focused on question 51 on the baseline survey which asked the respondent if they had ever taken a bone density test to check for osteoporosis and question 59 which queried the respondents about their educational background.

### 2.2. Study Population

**Figure 1:**
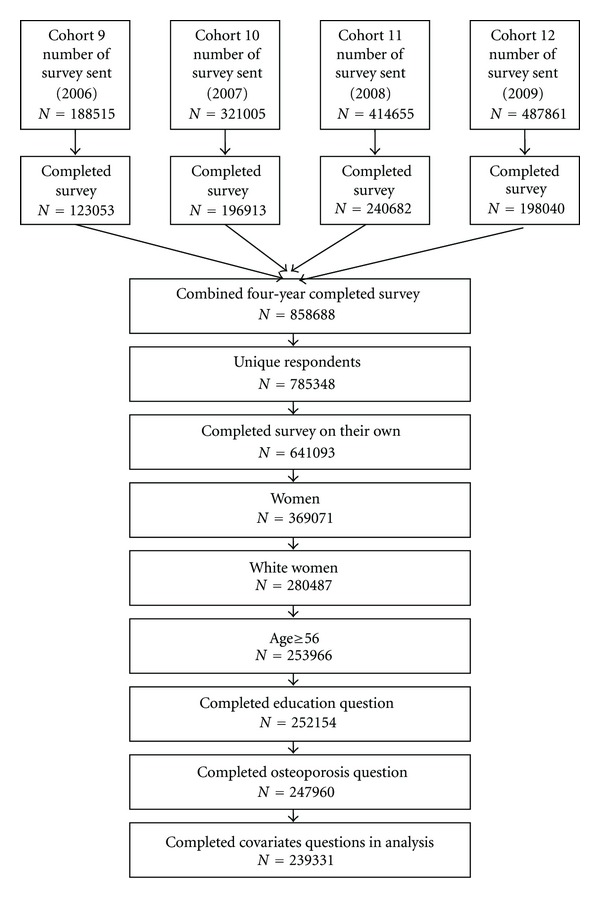
Data pool selection process (flow chart).

This paper used HOS cohorts 9 through 12 baseline surveys collected from 2006 through 2009, respectively, [20–23]. Each cohort of the HOS survey contained Medicare managed plan recipients accounting for about 10% of Medicare recipients of all ages, races, and gender. Since the target population of this paper was white elderly females on Medicare managed care who provided information on all of the variables we used (listed in the next section), a few modifications to the sample pool were made. The subject flow is shown in Figure 1. A total of 858,688 persons completed surveys in all four years, from 2006 to 2009. Some of the respondents completed multiple surveys during these four years, but only the first survey each respondent completed was chosen for this analysis. The number of unique respondents was 785,348. Many respondents did not complete the HOS survey on their own (which might be a surrogate for being in a long-term care facility or otherwise not able to make medical decisions) and were thus taken out to obtain a cognitively intact group of 641,093. Out of these 641,093 surveys, 369,071 were of women, 6.2% of whom did not report race. Of those 346,378 who reported race, 81.0% (or 280,487) were white.

Of the 280,487 white women who responded to the HOS survey on their own, 26,521 were below 65 years old and therefore excluded. Of the remaining 253,966 elderly white women, 14,635 did not answer at least one of the questions which were analyzed in this study and therefore were excluded, leaving the final analysis sample size of 239,331.

### 2.3. Variables

The two main variables in this study were whether or not a respondent ever had a bone density test and the level of education classified into three groups: less than a high school education, completed high school with no further education, and more than a high school education. Whether or not a respondent ever had a bone density test was asked as “*have you ever had a bone density test to check for osteoporosis, sometimes thought of as “brittle bones”? This test may have been done to your back, hip, wrist, heel or finger*.” 

Six other variables (age, body mass index (BMI), marital status, smoking, year of survey, and region) were also evaluated as either potential confounders or as indicators of further need to have a bone density test. In order to maintain the confidentiality of the respondents, HOS collapsed certain variables into categories. The age of every respondent was collapsed into two groups, 65 to 74 or 75 and above; marital status was collapsed into either currently married or currently nonmarried, and BMI was collapsed into either BMI ≥ 30 (obese) or BMI<30. Smoking was evaluated using the question “*do you now smoke every day, some days, or not at all?*” with smokers being classified by the HOS dataset as respondents who responded “*every day*” or “*some days*” and nonsmokers being classified as those who responded “*not at all*.” The year of survey was classified as cohort 9 (2006) through cohort 12 (2009) following the format HOS used. Finally, geographical region where the respondents received their medical treatment was recorded as a number between one through ten based on the Standard Federal Region used by the Center for Medicare and Medicaid Studied which is available at the CMS website [24].

The specific regions 1 through 10 were the following: New England, New York, Mid Atlantic, Southeast, Great Lakes, South Central, Midwest, Mountains and Plains, Pacific Southwest, Pacific Northwest [20–23].

### 2.4. Statistical Analysis

Descriptive statistics summarized respondent characteristics (age, marital status, etc.) in each education group (less than high school, completed high school with no further education, and more than high school). Percentages of respondents reported ever having a bone density test was summarized within all respondents, and within each education group. Univariable logistic regression examined the relationship between women having ever taken the bone density test and their education level, age, BMI, marital status, smoking, year of survey, and region. The three education levels, less than high school, completed high school with no further education, and more than high school were coded into two dummy variables with the education level of less than high school being used as a reference group. Afterwards, a multiple logistic regression analysis was performed to examine the relationship between women taking the bone density test and education levels adjusting for other characteristics. Given the large sample included in this analysis, 99% confidence intervals were provided, and a *P* value less than 0.01 was considered as statistically significant.

## 3. Results

Characteristics of responders are summarized in Table 1(a). Of the 239,331 total respondents, 45,358 (19.0%) reported having less than a high school education, 104,446 (43.6%) having completed high school with no further education, and 89,527 (37.4%) having more than a high school education. The population distribution for the other variables are available on Table 1(a). Compared to those who completed high school but had no further education, respondents with less than a high school education were more likely to be older, be currently single, have a BMI 30 or above, and smoke. Meanwhile, compared to those who completed high school with no further education, the respondents with more than a high school education were more likely to be younger, be married, have a BMI less than 30, and not smoke.

Percentages of responders reported taking bone density test are summarized in Table 1(b). Overall, 74.4% reported they had taken the bone density test for osteoporosis at some point. Within respondents with less than a high school education, 62.7% reported to have taken a bone density test while 73.8% of respondents with just a high school education and 81.0% of the respondents with more than a high school education reported having taken the test (*P* < 0.001 for equality among education groups). Although the three education groups had slight differences in demographic characteristics, the trend for more education being correlated to a higher percentage of respondents tested persisted all at *P* < 0.001 within each age, BMI, marital status, smoking status, and survey cohort strata (Table 1(b)). It is worth noting that the percentage of people who reported having the bone density test increased 1%-2% each year, from 70.9% in 2006 to 76.8% in 2009 (*P* < 0.001). The annual increase in percentage of responders having bone density test over time was similar across all education levels.

The logistic regression results are shown in the bottom half of Table 1(b). The univariable logistic regression showed that the odds ratio of taking the bone density test for respondents with a high school education versus respondents with less than a high school education was 1.68 (99% CI: 1.63–1.73). Meanwhile, the odds ratio of taking the bone density test for respondents with more than a high school education versus those with less than a high school education was 2.54 (2.46–2.63). Univariable logistic regression also showed a positive associations between taking the bone density test and being younger, having a low BMI, being married, and not smoking as shown on Table 1(b).

When all six characteristic variables were included in a multivariable logistic regression, the adjusted odds ratio of taking the bone density test for respondents with a high school education versus respondents with less than a high school education was 1.61 (99% CI: 1.56–1.66) which was almost the same compared to the univariable model. The adjusted odds ratio for more than a high school education versus less than a high school education was 2.39 (99% CI: 2.31–2.47). The positive associations between having the bone density test done and the person being younger, nonobese, currently married, not smoking, and with later calendar year for the HOS survey in the multivariable model all were consistent with those in the univariable models.

## 4. Discussion and Conclusion

There was a consistent positive association between a respondent's having a higher level of education and having ever taken the bone density test in elderly white women receiving managed Medicare. Overall almost 20% more elderly white women with beyond a high school education had a bone density test compared to those with less than a high school education (81.0% versus 62.7%), with those who had completed high school with no further education being somewhat in the middle (73.8%). Even after adjusting for potential confounding factors of age, body mass index, marital status, smoking status, time of survey, and region, those with higher education levels were independently more likely to have taken the bone density test. Lower education was directly associated and might be causal in the nonuse of bone density testing, as the odds ratios between education level and having bone density testing did not differ greatly between the univariable models and the multivariable model which adjusted for demographic and needs variables (to the level such variables were available in the HOS). It is important to note that these women were all in managed care plans that ostensibly should be emphasizing preventive care including elimination of disparities. Therefore, the association of lower education with nonuse of bone density testing might be even greater among elderly white women who are not in managed care.

While bone density testing in elderly white women increased with calendar time, penetrance was still well below 100% in this high-risk group of women receiving managed care, particularly for those with lower education. Some physicians may not have recommended osteoporosis screening for all eligible women despite published guidelines, since there is no direct evidence that screening BMDs reduce the community burden of fracture; however, those recommendations would not necessarily differ for patients of different educational status. As a disparity in use of preventive services, a patient's lack of education may be problematic to overcome both because it may hinder communication and understanding and because it may not be as easily recognized by the provider as are other disparities such as gender and race.

Among the other variables considered in this study, smoking, obese, and currently single persons were less likely to have had bone density test. This is problematic as smoking is a risk factor for osteoporosis and single persons may have less immediate support if experiencing a bone fracture. Smoking, obesity, and perhaps being single may reflect consequences of characteristics that limit one's ability to control health behaviors including seeking a bone density test. To that end, women with less education were more likely to smoke, be obese, and be single which is consistent with the previously noted associations of low education and health disparities.

One strength of this study is the large sample size. With almost 240 thousands total respondents, the smallest subset of the population stratified by the interested variables still consisted of more than 1000 respondents. Weaknesses of this study are that certain risk factors for osteoporosis such as alcohol usage, years since menopause, prior medical history, and physical activity were not collected in the public HOS database and therefore not analyzed. BMI was analyzed; the HOS dataset only reported obesity if a respondent's BMI was above or below 30. However, the risk factor for osteoporosis is having a low BMI below 19 [25]. Therefore, the available BMI data could not fully capture the effects which low BMI had on the use of osteoporosis testing. Without these data points, we did not have sufficient individual-level data on fracture risks to calculate FRAX scores, or to determine whether patients were started on osteoporosis treatment without referral for BMD screening based upon history of prior fracture or other risk factors.

Also, this study was restricted to managed Medicare patients, but one might expect that educational disparities would be even larger in the general Medicare population or among nonwhites. Still the amount and type of insurance of each respondent was not collected and analyzed. This is important because the BMD test is only fully covered by Medicare under certain conditions[20]. Medicare only covers patients at risk for osteoporosis as determined by their doctors and patients still need to pay for 20% of the testing fee [26]. Thus, social and economic factors such as income, location of residence (e.g. urban, suburban, rural), and proximity to friends and loved ones, which were also not collected, could also affect the percentage of people tested. The only such region variable available in the HOS dataset was location of residence by CMS Federal Regions which, although useful, did not capture the social economic factors which are of concern. However, Neuner [27] did not find any correlation between bone density testing and social economic factors in hip fracture patients.

Another potential weakness in this study is the lack of data regarding whether the physicians who provided care to each of the respondent recommended a BMD test to the respondents. It is possible that respondents with lower education were more likely to have a comorbidity which was more pressing than osteoporosis screening when they visited their physicians. Although we accounted for many different chronic comorbidities, there were many acute illnesses and some other chronic illnesses which we did not have data on. One last potential weakness in this study is the fact that only respondents who filled out every question pertinent to the study were included. This could cause problems because people in the lower education group were more likely to leave questions blank and therefore not be included in the study. However, the differences between the percentages of respondents excluded in each education group due to missing variables were all about one percent (data not shown) and extremely unlikely to effect the overall results of the paper.

In conclusion, we find strong and persistent disparities between lower education and bone mineral density testing among elderly white women in managed care. Further studies should be done to analyze how economic and social factors influence the disparity in osteoporosis testing as well as to find ways to close this education disparity gap. Denberg et al. have already done a study showing that a patient recall intervention can help increase the rate of osteoporosis testing [28]. Levy [29] also had success improving testing rates using chart reminders for physicians and mailing patients education information. It is unclear if these interventions will perform equally well across all education levels. A next step after identifying factors that cause low-education elderly women to not take a bone density test could be to implement direct intervention efforts towards those factors.

## Figures and Tables

**Table 1a:** (a)

	Characteristic by education level^a^			
	Total	Less than high school	High school	More than high school
Variable	(*N* = 239331)	(*N* = 45358)	(*N* = 104446)	(*N* = 89527)
Age				
65–74	57.2%	48.7%	56.8%	62.1%
75+	42.8%	51.3%	43.2%	37.9%
Body mass index				
Not obese BMI < 30	73.7%	70.4%	73.0%	76.4%
Obese BMI ≥ 30	26.3%	29.6%	27.0%	23.6%
Marital status				
Married	46.6%	38.8%	48.4%	48.4%
Nonmarried	53.4%	61.2%	51.6%	51.6%
Smoking status				
Every or some days	9.0%	11.6%	9.1%	7.4%
Not at all	91.0%	88.4%	90.9%	92.6%
Cohort				
9 (2006)	18.0%	19.0%	18.5%	16.9%
10 (2007)	24.6%	24.6%	18.5%	24.3%
11 (2008)	26.1%	25.7%	18.5%	26.7%
12 (2009)	31.3%	30.6%	18.5%	32.1%
Region				
(1) New England	5.0%	5.5%	5.1%	4.6%
(2) New York	10.9%	13.5%	10.7%	9.9%
(3) Mid-Atlantic	9.6%	10.5%	11.0%	7.6%
(4) Southeast	15.3%	17.4%	14.8%	14.9%
(5) Great Lakes	19.8%	18.4%	22.4%	17.5%
(6) South Central	7.8%	8.9%	7.3%	7.8%
(7) Midwest	6.8%	6.4%	7.2%	6.7%
(8) Mountains and Plains	4.0%	2.6%	3.5%	5.3%
(9) Pacific Southwest	11.5%	10.1%	9.9%	14.0%
(10) Pacific Northwest	9.2%	6.8%	8.1%	11.8%

^a^all *P* values < 0.001.

**Table 1b:** (b)

	Percentage of people having bone density test^a^			
	Total	Less than high school	High school	More than high school
Variable	(*N* = 239331)	(*N* = 45358)	(*N* = 104446)	(*N* = 89527)
All Subjects	74.4%	62.7%	73.8%	81.0%
Age				
65–74	76.9%	64.6%	75.9%	83.0%
75+	71.0%	60.8%	71.1%	77.8%
Body mass index				
Not obese BMI < 30	75.9%	63.8%	75.1%	82.4%
Obese BMI ≥ 30	70.3%	60.0%	70.5%	76.5%
Marital status				
Married	78.0%	66.0%	77.1%	84.0%
Nonmarried	71.2%	60.6%	70.8%	78.2%
Smoking status				
Every or some days	64.2%	55.1%	64.1%	71.5%
Not at all	75.4%	63.7%	74.8%	81.8%
Cohort				
9 (2006)	70.9%	59.6%	69.9%	78.5%
10 (2007)	73.3%	61.5%	73.2%	79.5%
11 (2008)	75.0%	63.2%	74.3%	81.5%
12 (2009)	76.8%	65.0%	76.3%	83.0%
Region				
(1) New England	80.8%	71.5%	80.5%	86.8%
(2) New York	76.6%	66.9%	76.5%	83.3%
(3) Mid-Atlantic	71.5%	57.4%	71.1%	82.1%
(4) Southeast	76.5%	64.9%	76.9%	83.0%
(5) Great Lakes	73.7%	61.6%	73.1%	81.1%
(6) South Central	73.6%	61.7%	73.4%	80.6%
(7) Midwest	75.8%	61.9%	75.1%	83.4%
(8) Mountains and Plains	73.5%	59.3%	70.8%	79.0%
(9) Pacific Southwest	71.9%	59.4%	70.8%	77.4%
(10) Pacific Northwest	72.6%	60.4%	70.6%	77.7%

	Logistic regression analysis			
	Univariable		Multivariable^b^	
Variable	OR (99% CI)		OR (99% CI)	

Education level				
HS = 1 versus less HS = 0	1.68 (1.63, 1.73)		1.61 (1.56, 1.66)	
More HS = 1 versus Less HS = 0	2.54 (2.46, 2.63)		2.39 (2.31, 2.47)	
Age				
65–74 versus 75+	1.36 (1.33, 1.40)		1.32 (1.28, 1.35)	
Body mass index				
BMI < 30 versus BMI ≥ 30	1.33 (1.29, 1.36)		1.39 (1.35, 1.43)	
Marital status				
Married versus nonmarried	1.43 (1.40, 1.47)		1.28 (1.24, 1.31)	
Smoking status				
No versus yes	1.72 (1.78, 1.64)		1.75 (1.82, 1.67)	
Cohort (reference = cohort 9)				
Cohort 10 versus cohort 9	1.13 (1.09, 1.17)		1.13 (1.09, 1.17)	
Cohort 11 versus cohort 9	1.23 (1.19, 1.28)		1.24 (1.19, 1.29)	
Cohort 12 versus cohort 9	1.36 (1.31, 1.41)		1.35 (1.30, 1.40)	

^a^All *P* values < 0.001.

^b^Multivariable model including education, age, body mass index, marital status, smoking status, cohort, and region.
